# Characterization of carrier behavior in photonically excited 6H silicon carbide exhibiting fast, high voltage, bulk transconductance properties

**DOI:** 10.1038/s41598-021-85275-6

**Published:** 2021-03-25

**Authors:** S. E. Sampayan, P. V. Grivickas, A. M. Conway, K. C. Sampayan, I. Booker, M. Bora, G. J. Caporaso, V. Grivickas, H. T. Nguyen, K. Redeckas, A. Schoner, L. F. Voss, M. Vengris, L. Wang

**Affiliations:** 1 Lawrence Livermore National Laboratory, 7000 East Avenue, Livermore, CA 94551 USA; 2 Opcondys Incorporated, 600 Commerce Court, Manteca, CA 95336 USA; 3 Department of Electrical Sciences and Engineering, Kyoto University, Kyoto, Japan; 4 Institute of Photonics and Nanotechnology, Vilnius University, Vilnius, Lithuania; 5 Laser Research Center, Vilnius University, Vilnius, Lithuania; 6 Ascatron II-VI, Electrum 207, Isafjordsgatan 22 (B, Plan 5), 16440 Kista, Stockholm Sweden

**Keywords:** Engineering, Optics and photonics

## Abstract

Unabated, worldwide trends in CO_2_ production project growth to > 43-BMT per year over the next two decades. Efficient power electronics are crucial to fully realizing the CO_2_ mitigating benefits of a worldwide smart grid (~ 18% reduction for the United States alone). Even state-of-the-art SiC high voltage junction devices are inefficient because of slow transition times (~ 0.5-μs) and limited switching rates at high voltage (~ 20-kHz at ≥ 15-kV) resulting from the intrinsically limited charge carrier drift speed (< 2 × 10^7^-cm-s^−1^). Slow transition times and limited switch rates waste energy through transition loss and hysteresis loss in external magnetic components. Bulk conduction devices, where carriers are generated and controlled nearly simultaneously throughout the device volume, minimize this loss. Such devices are possible using below bandgap excitation of *semi-insulating* (SI) SiC single crystals. We explored carrier dynamics with a 75-fs single wavelength pump/supercontinuum probe and a modified transient spectroscopy technique and also demonstrated a new class of efficient, high-speed, high-gain, bi-directional, optically-controlled transistor-like power device. At a performance level six times that of existing devices, for the first time we demonstrated prototype operation at multi-10s of kW and 20-kV, 125-kHz in a bulk conduction transistor-like device using direct photon-carrier excitation with below bandgap light.

## Introduction

The emerging global electric grid promises better integration of renewable energy sources, higher system stability, and minimized fossil-fuel use, potentially slowing the worldwide trend in greenhouse gas production^1,2^. 40% of consumed energy in the United States will be controlled with solid-state power devices by 2030 with “smart grids” potentially reducing CO_2_ production by approximately 18%^3,4^. Pulsed electric fields (PEF) have also been shown effective as a low energy, non-thermal, inactivation method of both viral and bacterial pathogens^5,6^. Efficient solid-state switching devices are required for both. Bulk conduction, high-voltage, photoconductive devices, where carriers are simultaneously generated and controlled throughout the device volume, minimize loss. After a detailed study of carrier dynamics, we demonstrated a multi-10s of kW, 20-kV, 125-kHz bulk conduction transistor-like device using direct photon-carrier excitation with below bandgap light using *semi-insulating* (SI) SiC single crystals^7^. Tailored recombination and weak optical absorption enable bulk conduction control^8^. In our study, we explored carrier dynamics with a 75-fs single wavelength pump/supercontinuum probe and modified transient spectroscopy. Results showed that a new class of efficient, high-speed, high-gain, bi-directional, optically controlled transistor-like power device is possible.

**Figure 1:**
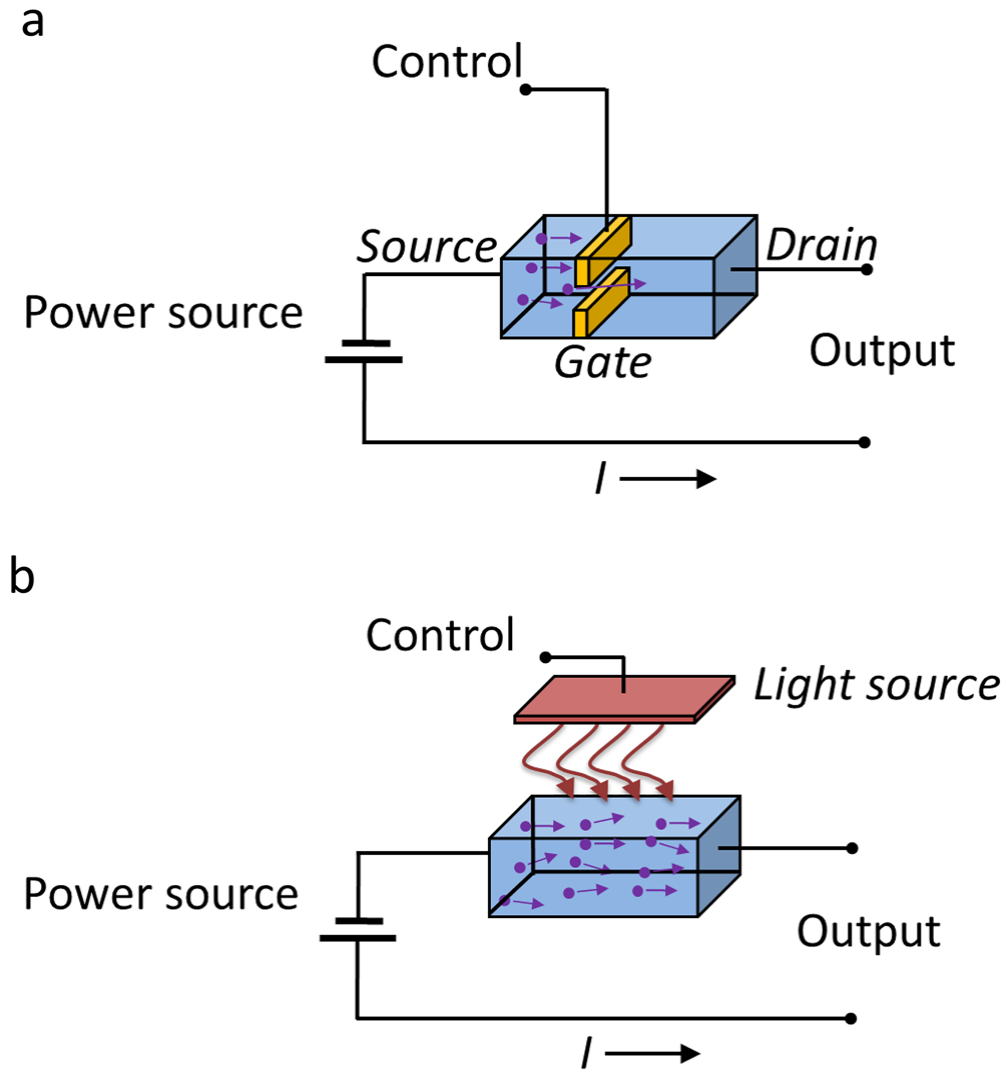
Highly simplified junction field effect control contrasted with bulk photonic control. (**a**) Charge carriers flow from the source to drain and are controlled by the field at the gate. The time delay from the action at the gate to a response at the drain is limited by the carrier drift speed. The slower the response, the greater the energy wasted. Poorer energy efficiency and difficulty in stacking to high voltage DC (HVDC) transmission result because of gate isolation requirements. (**b**) Direct control of carriers in the bulk by photons using below bandgap excitation creates carriers instantaneously in the bulk. The time delay from the action at the optical source to a response at the output is limited only by the light source and the conglomerative effects of the recombination dynamics. Greater energy efficiency and the ability to stack to HVDC result from the light isolation.

In a highly simplified field effect solid state transistor (Fig. 1a), when a voltage is applied between two opposing terminals (for instance a “source” and “drain”), a third intervening terminal (e.g., a “gate”) can be used to control the current, ***I***, through the device. The action at the control terminal is delayed at the drain by the carrier transit time. For illustrative purposes, this delay is proportional to ***x/µE***, where ***x*** is the drift region length that supports the high voltage, ***µ*** is the carrier mobility, and ***E*** is the applied electric field^9,10^. As a result, scaling device speed and high voltage capability are mutually exclusive: thick drift regions are required to achieve adequate voltage capability but result in increased carrier transit times and slow transition speed. Even with state-of-the-art SiC junction devices with material breakdown limits of ~ 3-MV/cm, intrinsically limited carrier drift speed (< 2 × 10^7^-cm-s^−1^), slow transition times (~ 0.5-μs) and limited switching rates (~ 20-kHz) at ≥ 15-kV are typical^11^. These limitations adversely impact efficiency through wasted energy from increased device transition time (i.e., off-to-on state or on-to-off state) and hysteresis loss in external magnetic components.

With direct generation and control of bulk carrier density by photons, however (Fig. 1b), drift dynamics are no longer relevant, and the approach is fundamentally limited only by the conglomerative effects of the recombination dynamics (~ ns time scales).

Junction and field effect transistors also have a defined property known as *transconductance* (i.e., “transfer conductance”) or ***g***_***m***_. The quantity relates the input to the output by the ratio ***(∆I/∆ξ***_***in***_***)***, where *ξ*_***in***_ is the controlling input voltage. Amplification is then ***V***_***out***_** = V**_***g***_*** g***_***m***_*** Z***_***o***_, where ***V***_***g***_ is the input and ***Z***_***o***_ is the load impedance^12,13^.

Past photoconductive switches did not focus on a potential transconductance like property, but rather on short pulse (< 100-ns) megawatt peak power levels, latch-on bi-modal on–off switching, or required the use of dual optical energies to initiate and then de-latch the device by quenching carriers^14–18^.

But for this new device, from a highly simplified continuity equation in the bulk of the crystal, with a source term $\frac{{g}_{o}} S(t)$, a general solution of the conductivity is:

1 $${\varvec{g}}\left( t \right){\propto}e^{{ - \frac{t}{\tau }}} \mathop \smallint \limits_{0}^{t} S\left( {t^{\prime}} \right)e^{{\frac{t^{\prime}}{\tau }}} dt^{\prime},$$

where *τ*—recombination time, ${g}_{o}$—maximum conductivity, and *S(t)*—normalized optical intensity. With a prompt excitation impulse *δ(t)*, ***g(t)*** is an exponential decay. But for slowly varying *S(t)* compared to *τ*, ***g(t)***$\propto$* S(t)* or $\frac{1}{S\left( t \right)}$→*ξ*_***in***_. That is, bulk, extrinsic, photoconductive semiconductor control devices made from semi-insulating SiC and similar materials exhibit a transconductance-like property when the recombination time is small (~ ns) compared to the highest frequency components of the applied signal (~ 10s of ns in power electronics).

We exploit V doped semi-insulating (SI) SiC as it has been studied extensively as the base substrate for LED and device production. But the physics of the semi-insulating conduction process are unexplored and not fully understood due to the complexity of the defect structure^19^. As is well documented in the literature, V at the substitutional Si site forms deep mid-gap levels in most polytypes of SiC with amphoteric behavior (i.e., exhibit acceptor and donor states depending on the Fermi level^20^). V captures carriers from unintentional shallow impurities producing semi-insulating material^7^. During optical excitation, V sites become effective recombination/generation centers^21^. Electrons and/or holes (depending on wavelength) are excited to the conduction and valence bands, respectively, in the bulk. For a given light intensity, the excitation and recombination are in equilibrium to maintain a specific conductivity. When the light is removed, the carriers recombine in the bulk and the turn off speed is then limited only by their respective recombination time. By controlling non-radiative recombination (NRR) center density we trade fidelity and speed against equivalent power gain.

## Results

**Figure 2:**
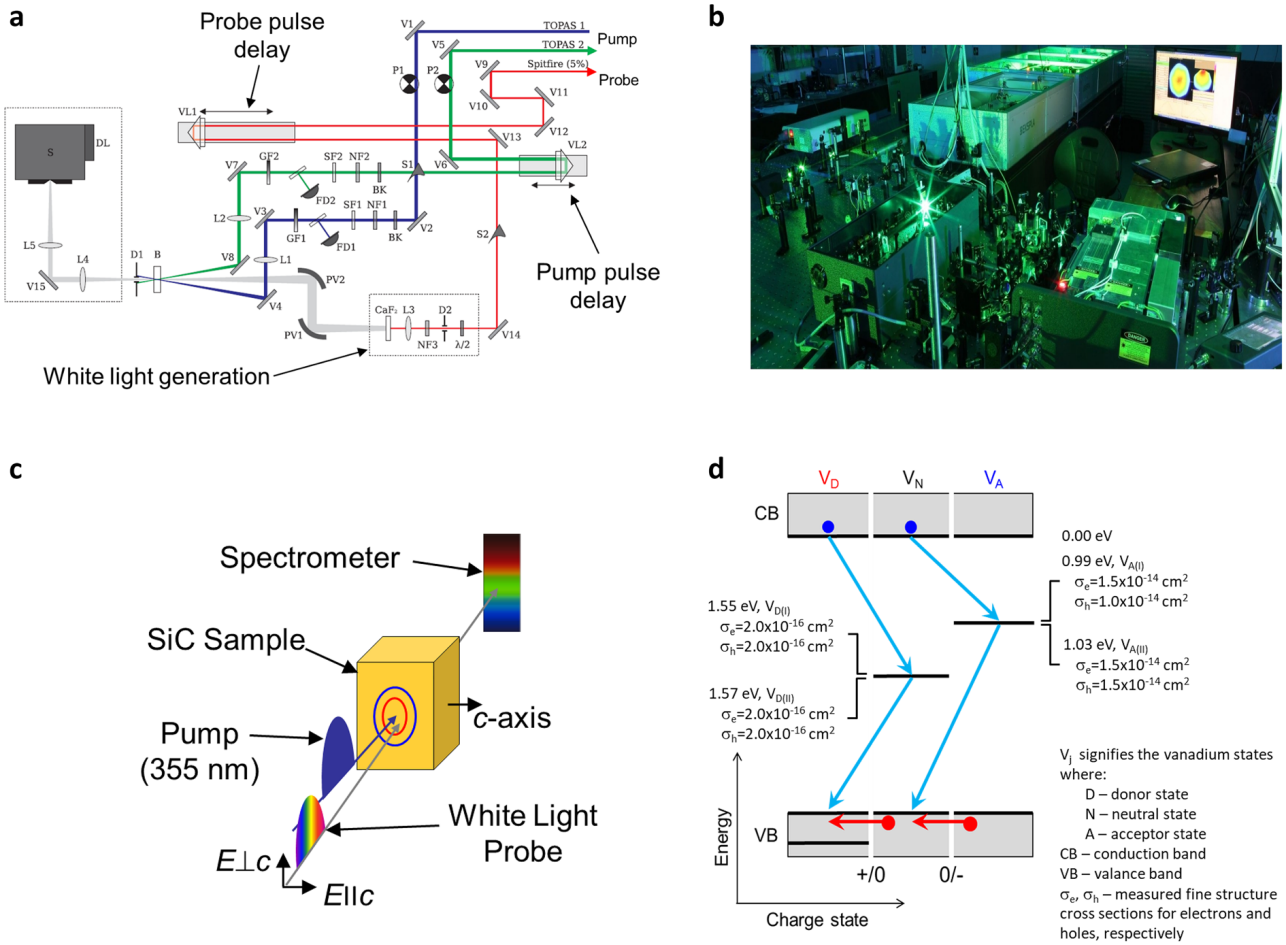

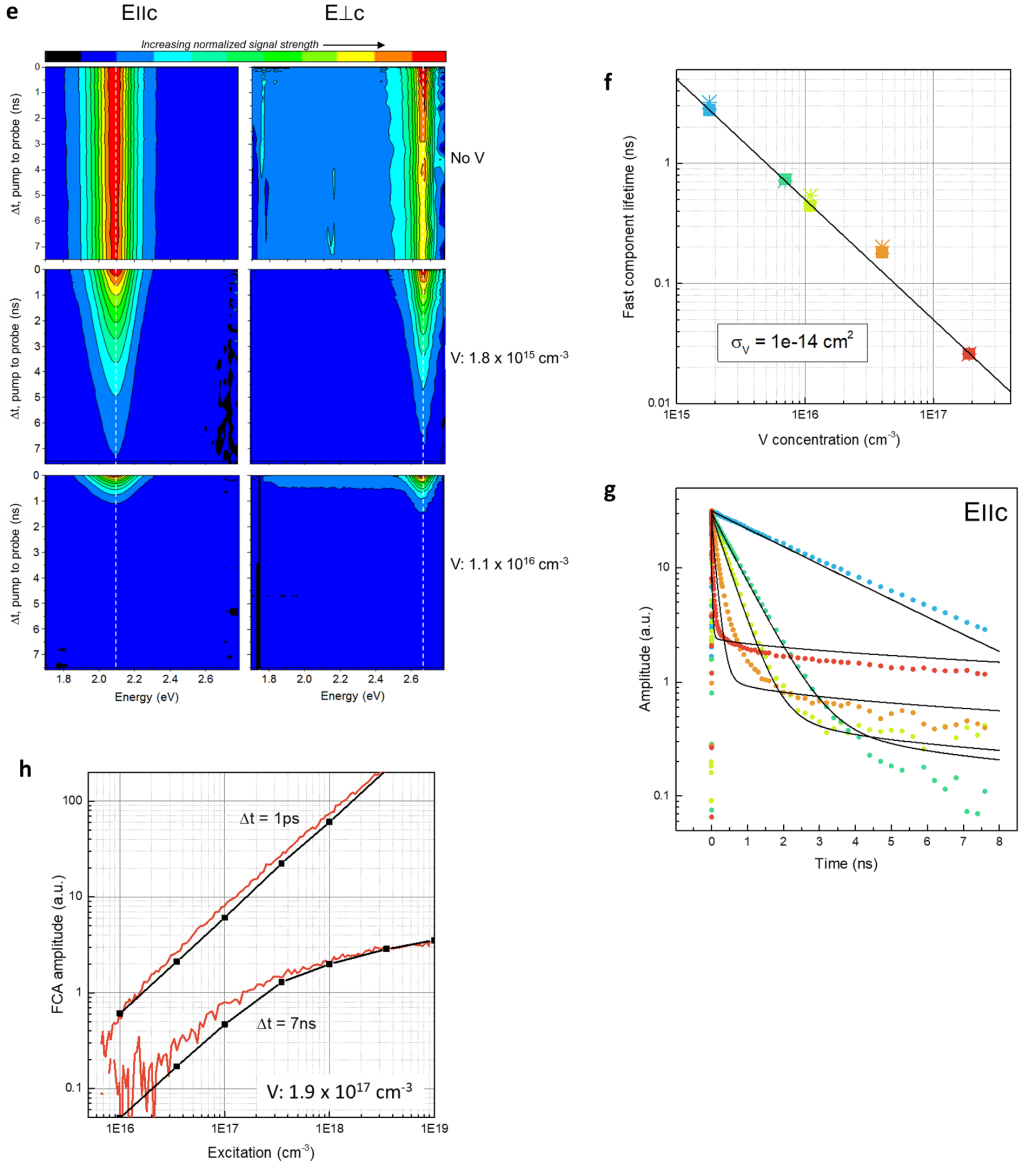
SI SiC carrier dynamics measurements. (**a**,**b**) 75-fs pulses were generated with a Ti:sapphire laser at the 800-nm fundamental wavelength. Pump wavelength was converted to 355-nm by a parametric frequency converter while probe wavelength was transformed to a white light continuum by a sapphire plate. Measurement geometry is shown schematically in (**c**); both beams were focused (d_spot_ ~ 100-μm) and near perpendicular to the sample surface. Probe beam polarization could be oriented either parallel (*E*∥*c*) or perpendicular (*E*⊥*c*) to the principal “c” crystal axis. (**d**) The resultant bandgap model shown schematically with our highly refined spectra and cross section measurements. We deviate from previous approaches by also including the charge state of the vanadium (see “Methods” section). By imposing the conditions of energy and charge conservation, the allowable transitions for electrons (blue) and holes (red) are also shown. This model serves as the basis of the target analysis of the data from which we can determine coefficients for a coupled set of first order rate equation^32^. (**e**) Time evolution of the GTA plot for different V concentrations shows the centroid remained constant for *E*∥*c* (2.08-eV) and *E*⊥*c* (2.66-eV). (**f**,**g**) summarize the fast and slow component data as a function of V concentration and the model fit and indicates a very prompt carrier excitation on the order of the excitation pulse width. V concentration is shown in Table 1. In (**g**), increased decay occurred at increased V concentration until about 10^16^-cm^−3^. At about that concentration level, a fast-initial decay with a long tail starts to emerge and becomes dominant at a V concentration of > 7 × 10^15^-cm^−3^. This result suggests that the standard Shockley–Read–Hall recombination model is inadequate, particularly with high V concentrations. (**h**) Shows the free carrier absorption (FCA) shortly after the initial pump (∆t = 1 ps) and at late time (∆t = 7 ns) and the model fits.

Characterization of carrier dynamics used a combination of ultrafast (75-fs) pump-white-light-probing with 3D global and target analysis (GTA) (Fig. 2a–c) and deep level transient spectroscopy (DLTS); global refers to simultaneous analysis of all measurements, target refers to the applicability of a specified model (specific details are provided in the “Methods” section)^22^. Deep levels, however, are difficult to measure accurately with published results varying by an order of magnitude^23^. The standard method using DLTS fills recombination centers with a pulsed electric field or pulsed light source (~ 20 ms). Carriers modify the volume capacitance. As emission occurs in the volume at some rate and at a specific temperature, a capacitance transient develops. By observing the transient resulting from thermal emission at different temperatures, the energy and cross section can be determined^24^.

For DLTS, we employed a modified method by varying the trap filling times from 50-ns to > 10-ms, used a combination of electrical and optical filling of the centers, and used the GS6 correlation functions^25,26^. Results did not show the typical variability of the previous literature and are presented in Fig. 2d with added detail on allowed transitions being detailed in the “Methods” section.

Two main recombination centers in SiC:V exist where the interplay between states depends on the excitation wavelength and concentration of V^27^. But we find that the classical trap assisted Shockley–Read–Hall (SRH) model describing the simple decay of carriers from the conduction band to the recombination center also required inclusion of the amphoteric nature of the V for the analysis^28,29^.

Commercial Lely grown substrates and a high-quality set of epitaxial SI 4H-SiC samples were developed specifically for carrier dynamics studies (Table 1). V concentration in the epitaxial material was controlled by the partial pressure of the precursor gas during growth. Concentration was verified by standard secondary ion mass spectrometry (SIMS). Bulk 6H-SiC is grown by the well-known modified Lely process, but high concentrations of unintended dopants (N, Al, B) can be introduced. As a near parallel material, we grew 4H polytype epitaxially with precise control of V and minimal unintended dopants (N ~ 1–3 × 10^14^-cm^−3^)^30,31^.

**Table 1:** Secondary ion mass spectroscopy (SIMS) substrate doping.

Sample	Color code	Concentration (10^15^ cm^−3^)	
		V	Residual dopants (n-p) type
Epi-0		–	–
Epi-1		1.8	0.9
Epi-2		7.0	1.4
Epi-3		11.0	0.9
Lely-1		40	19.5
Lely-2		190.0	17.9

Color code refers to data plotted in Fig. 2f,g

Ultrafast pump-white-light-probing was performed on all samples with the GTA 3D evolution shown in Fig. 2e. The 75-ps pulse width allowed sufficient intensity for the measurements while ensuring that two-photon pumping (threshold ~ 10^9^-W-cm^−2^) from the valance band was avoided.

Referring to the color code in Table 1 for vanadium concentration, Fig. 2f,g summarizes the fast recombination data as a function of V concentration. For SRH, the recombination time is inversely proportional to the cross section ($\tau_{TR} \propto 1/\sigma_{TR} N_{TR}$, where *σ*_*TR*_ and *N*_*TR*_ are the trap cross section and concentration, respectively). From these figures, it is clear that the decay for elevated V concentration is more complex than simple SRH recombination. A GTA fit using this assumption overlaid with the data shows agreement with the measured recombination times using the spectroscopy cross section for the V acceptor for the electrons (Fig. 2g). These two observations lead to the conclusion that the fast recombination channel is consistent with the classic SRH model.

Target analysis of the slow components is based on the amphoteric model using first order rate equation^32^. The data (Fig. 2g) and model results agree, albeit a better fit occurs at the minimum and maximum V doping. Finally, Fig. 2h shows the free carrier absorption (FCA) shortly after the initial pump (∆t = 1-ps) and at late time (∆t = 7-ns). At late time and high carrier injection densities (> 3 × 10^17^-cm^−3^), the FCA amplitude decreases indicating that the V centers are saturated. By applying the GTA and invoking an amphoteric model, a good fit of the data occurs leading us to conclude that an amphoteric model is necessary to understand our observations in SiC:V material.

**Figure 3:**
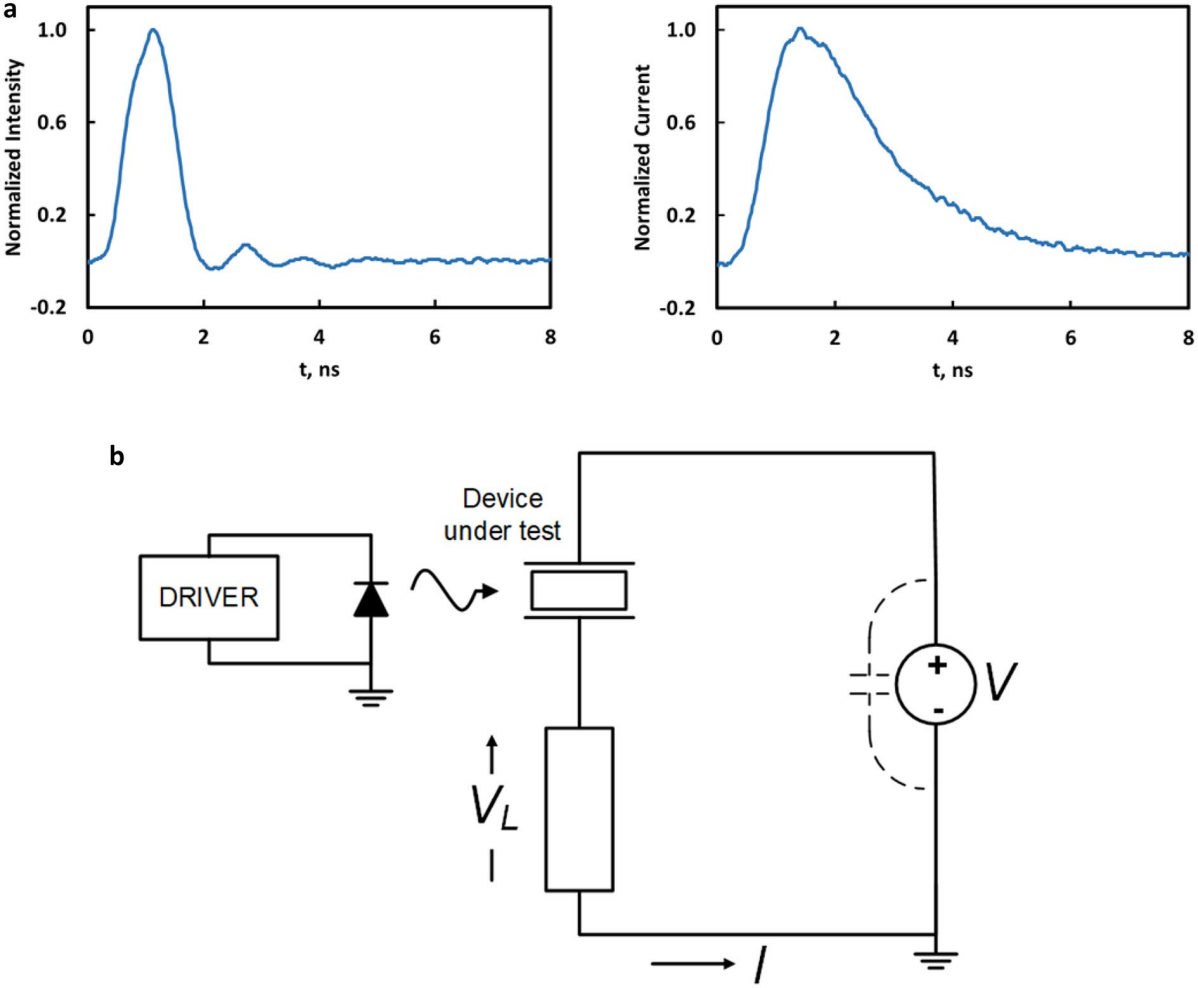

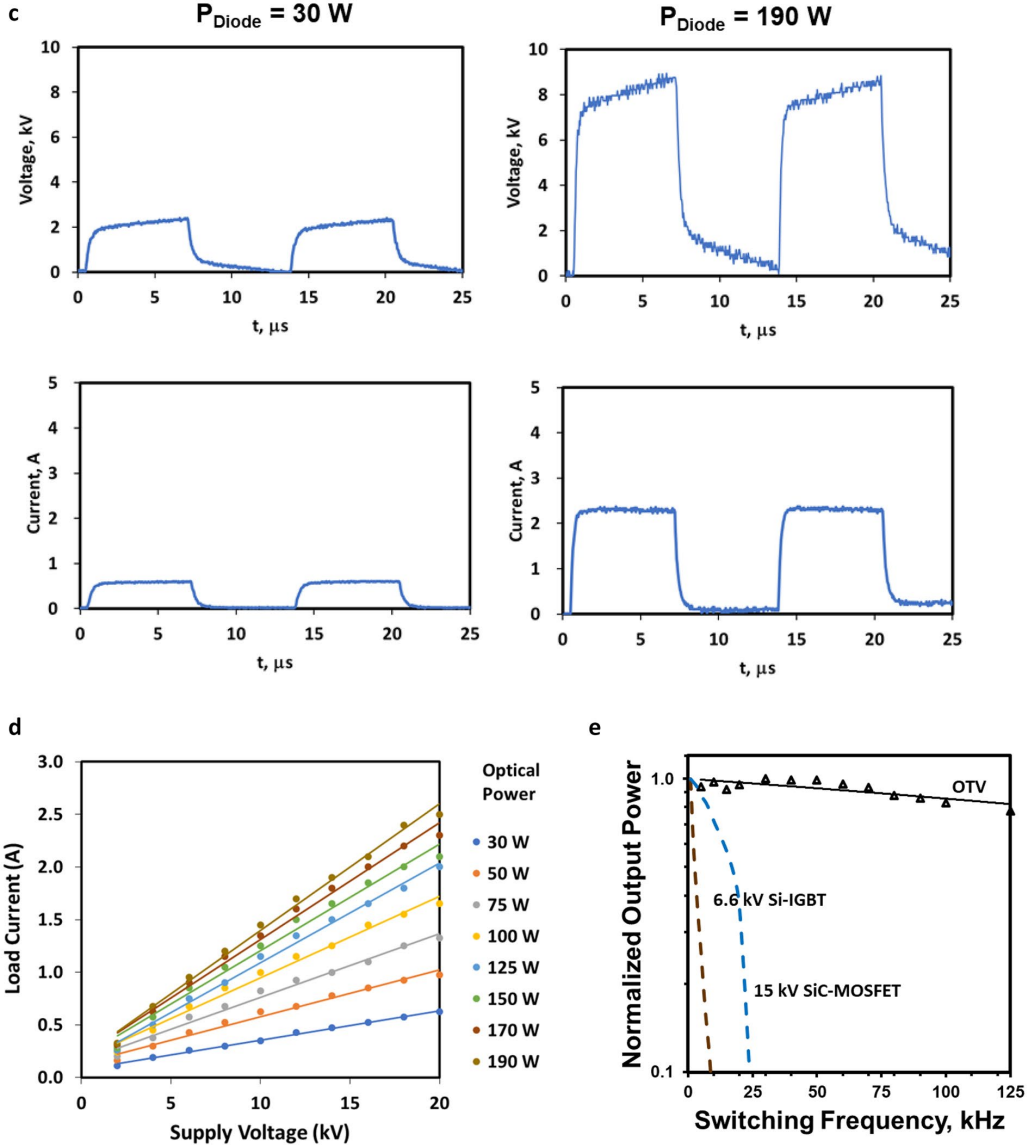
Results of a proof-of-concept test. (**a**) Electrical response of the OTV to an optical delta function of about 1 ns FWHM (left). Current waveform (right) demonstrates a fast-equivalent leading edge and decay based on carrier recombination (Eq. (1)). (**b**) 20 kV test circuit with utilizing a grounded load and electrically floating OTV. (**c**,**d**) Measurement of V_L_ and load current, demonstrating the “transconductance like” control similarly exhibited by classical junction devices. (**e**) Speed comparison of state-of-the-art high voltage junction devices with a prototype bulk conduction device. Switching frequency is significantly faster than MOSFET and IGBT technology, enabling high speed low loss switching. Comparison is based on reference^11^. Power is normalized to the output at 1 kHz. The slope in our bulk condition OTV device was due to external circuitry.

Several proof of concept *optical transconductance varistor* (OTV) devices based on this principle were built and tested. Response in a low inductance circuit is shown in Fig. 3a. Device rise-time relative to the applied laser pulse is essentially the same indicating a prompt excitation of carriers as we measured in the pump-probe experiments (Fig. 2g); a characteristic decay based on carrier recombination is clearly evident after the peak (Eq. (1)). Operation in this fast pulse mode was possible up to 1-MHz and was limited by the circuit elements.

Classic testing of a power device requires a grounded device, otherwise performance limiting isolation transformers or power-over-fiber (PoF) must be used. But with the OTV, the natural electrical isolation through the optical drive allowed a grounded load rather than a grounded device (Fig. 3b)^33^. Testing was done either with a copper-foil-strip-wound Metglas ribbon inductor, a resistive load, or a combination of the two. Photoexcitation was with a below bandgap, multi-hundred-watt commercial fiber coupled laser diode array driven with electronics to mimic gate operation of a MOSFET where the optical isolation enabled the OTV to float independently.

Figure 3c,d demonstrates the transconductance like control properties of a SI SiC bulk illuminated single crystal device. In Figure 3c, we are demonstrating the OTV as a switch as is generally used in power inverter applications; V_L_ is the load voltage and I, the load current (Fig. 3b). Figure 3c shows the variation for two optical powers with a summary of these characteristics over a wider range shown in Fig. 3d. Using *load-line analysis* with Fig. 3d, however, the amplification across an output load can be determined for a given optical power variation which is similar in operation to a transistor^34^. The device was subjected to in excess of 20-kV with a conduction current of approximately 2.5-A and up to a 125 kHz switching rate, well above the 20 kHz limitation of state-of-the-art devices. This particular detailed data presented was taken at 75-kHz switching frequency. Forward power gain was in excess of 20-dB (optical to electrical), nominally consistent with standard devices.

We also compared the switching frequency response with state-of-the-art high voltage junction devices^11^ (Fig. 3e). The triangles represent normalized data taken at corresponding frequencies. The slight downward slope is attributed to the circuit response. Classical high voltage junction devices are fundamentally limited above 20-kHz due to the slow carrier drift speed. In addition, for a constant on resistance, to compensate for the thicker drift region, the conduction area needs to be increased which results in increased capacitance. This increased capacitance shunts the switched current at high frequency, further limiting switching speed. Because the OTV exploits bulk illumination, it does not have this limitation.

## Discussion

Our modified method DLTS varying the trap filling times from 50-ns to > 10-ms and using a combination of electrical and optical filling of the centers yielded consistent results to the first and second significant place for the cross section and energy level. This more precise result is in contrast with our own non-modified techniques and the published literature that shows significant variation.

Characterization of carrier dynamics show two recombination channels. The first is consistent with the classic Shockley–Read–Hall (SRH) model; the second required our invoking an amphoteric model for explanation. For faster recombination times, by approximating the decay with a simple exponential, the effect can approximate a transconductance like behavior similar to transistor devices. By using near total internal reflection, the approach enables bulk conduction control that enables a new class of efficient, high voltage, high-speed, high-gain, bi-directional, optically controlled transistor-like power devices. Our simple prototype demonstrated operation at multi-10s of kW and 20-kV, 125-kHz and for the first time demonstrated bulk conduction transistor-like control using direct photon-carrier excitation with below bandgap light. This prototype performance level is six times faster than existing solid-state devices.

Fully adopted, based on switching speed alone, we estimate CO_2_ reductions by > 10% worldwide at 50 kHz. Further improvements to the technology include better vertical integration and operating the device with total internal reflection to decrease on resistance (~ 1 Ω) and increase optical efficiency (> 20 dB)^8^. Thus, with the potential of inexpensive laser diode sources (~ $0.03/peak-watt per the National Academy of Sciences^35^) optically based bulk conduction devices using near total internal reflection for increased forward power gain, may someday substitute for increased efficiency junction devices in high voltage power electronics.

## Methods

A complete set of > 40 samples were characterized. Because the V dopant and other unintended dopants such as N, B, or Al can vary during bulk growth, multiple Secondary Ion Mass Spectrometry (SIMS) measurements were required over the substrate surface. High-quality epitaxial materials were grown by the CVD technique (“Epi” label, Table 1) in collaboration with Ascatron II-VI, Inc. The 250-μm epilayers were grown on the 4° off *n*-type substrates with V doping ~ 1 × 10^16^-cm^−3^. The remaining substrates were obtained commercially from various manufacturers.

Substrates were diced parallel to the c-axis into 0.5–1.0-mm bars. Epi wafers were stacked and temporarily bonded epi-side-to-epi-side to avoid damage during the dicing operation. All samples were subsequently lapped then characterized mechanically.

For device fabrication, successively finer diamond grits starting from ~ 10-μm were used as an initial step on a planetary lapper. For each face, the substrates were temporarily bonded to glass optical flats, then were fixtured to size the substrates in the remaining planes. All planes were subsequently chemical mechanically polished using a Strasbaugh polisher with an eccentric overarm. A gold coating on the faces provided electrical contact.

Ultrafast pump and probe pulses of 50–75-fs duration were generated by a Ti:sapphire laser (Coherent Libra-USP) at 800-nm. Pump wavelength was converted to 355-nm by a parametric frequency amplifier (LightConversion Topas-800) while probe wavelength was transformed into a white light continuum by propagating the beam along a sapphire plate (Fig. 2a,b). Both beams were focused almost perpendicular to the sample surface at ~ 2° angle with respect to each other. The ~ 60-μm diameter probe was aligned in the middle of the ~ 100-μm diameter pump (Fig. 2c). The geometry allowed probing with the light polarization parallel (*E*ǀǀ*c*) or perpendicular (*E*⊥*c*) to the principal axes. The transmitted probe beam was directed through a spectrometer-diode array. Full scans of the samples required approximately two hours.

To determine the bandgap states, energy levels, and cross-sections, we used an improved DLTS measurement that is described in greater detail elsewhere^36^. In standard DLTS, the space charge region underneath the Schottky barrier (or p–n junction) is expanded and collapsed (i.e., the filling pulse). Traps whose energy levels drop below the Fermi level (n-type as an example) during the filling pulse capture electrons. Once a space charge region is re-established and the effect of the electric field forces the trap energy levels above the Fermi level, electrons are emitted. The emission rate is a function of temperature and the emission process; the process results in a temperature dependent capacitance transient^24,37^.

For analysis, the measured capacitance transient is multiplied by a correlation function W(t) defined over time interval [*t*_*d*_ to *t*_*c*_] and integrated:

2 $$S\left[ {t_{c} ,t_{d} } \right] = \frac{1}{{t_{c} }}\mathop \smallint \limits_{{t_{d} }}^{{t_{d} + t_{c} }} \Delta C\left( t \right)W\left( t \right)dt.$$

The outputs for different specified emission rates are then plotted as a spectrum over temperature. The peaks are related to deep levels in the material and their position and shape depends on the trap energy level and capture cross-sections.

The shape of the weighting function determines selectivity, resolution, and sensitivity to the signal-to-noise ratio. The main advantage of using correlation functions over other methods in the analysis is the insensitivity to baseline offset^38^. In the case of very low signal levels, a low-resolution cosine correlation function is used. Here, the GS*n* function, based on the Gaver–Stehfest numerical Laplace transform inversion algorithm offers the best trade-off between signal-to-noise sensitivity and selectivity. Our focus here is the GS6 function because of the improved resolution capability. But the function is very sensitive to noise in the transient^25,26^.

The samples for DLTS were typically mounted onto aluminum-oxide plates with AgPd contact fingers using Ag-paste. Bonded Al wires connected evaporated diodes to the contact fingers. The sample was mounted on a small thermo-chuck, which included a heating cartridge and an RTD temperature detector. The assembly could be lowered into a liquid nitrogen bath for cooling. This setup allowed unhindered optical access to the sample and provided temperature stability of up to ± 0.1° K. An Agilent 33220A or Tabor 8024 pulse generator provided electrical pulses down to 50-ns length. The capacitance transient measurement was done with a Boonton 7200 capacitance bridge.

For optical DLTS (O-DLTS), the excitation source was typically a 355-nm Nd:YAG passively Q-switched laser. A fast photodiode provided a post-pulse trigger for an Agilent U1082A digitizer/oscilloscope. The laser focused onto the sample by a lens at a 72° angle to the sample surface. A Nikon 20× microscope objective collected, parallelized, and directed photoluminescence onto a Hamamatsu PMT. Transients were averaged for up to 30-min. at high temperatures, ultimately giving measurements with a dynamic range of at least 4 orders-of-magnitude.

The simplest and most widely used parameter extraction method used in DLTS are Arrhenius plots. Standard use of a Gaussian function to fit the DLTS peak and extract the peak center temperature sometimes delivers erroneous results when peaks overlap in the spectrum. The traditionally used correlation functions, such as sine, cosine or rectangular lock-in functions, also provide poor resolution, making peak separation difficult. Laplace-DLTS provided superior results. In this method, a numerical inverse Laplace transform is taken in order to separate different emission rates.

Another improvement over standard DLTS is multi-spectrum fitting using the higher resolution correlation function GS6. Here, several measurements made with different filling pulse lengths are fitted together using the same set of parameters to numerically simulate the entire DLTS spectrum. The peak heights are determined directly from the temperature dependent capture cross-sections and the filling pulse length. A numerical calculation of slow capture and re-emission in the transition region of the space charge region is also included in the model which allows an extended range of filling pulse lengths which can be used for the technique. Final results are shown schematically in Fig. 2d.

Quantitative correlation between the detected temporal and spectral changes was extracted using the global and target analysis (GTA) based on linear rate kinetics^22^. We use the modeling methodology outlined and similar to that presented by Klein^39^. From Fig. 2d we used a set of differential equations:

3 $$\begin{gathered} \frac{d\Delta n}{{dt}} = G - R_{DN} - R_{NA} , \hfill \\ \frac{d\Delta p}{{dt}} = G - R_{ND} - R_{AN} , \hfill \\ \frac{{d\Delta V_{A} }}{dt} = R_{NA} - R_{AN} , \hfill \\ \frac{{d\Delta V_{D} }}{dt} = R_{ND} - R_{DN} , \hfill \\ \frac{{d\Delta V_{N} }}{dt} = R_{AN} + R_{DN} - R_{NA} - R_{ND} , \hfill \\ \end{gathered}$$

4 $$\begin{gathered} R_{{DN}} = \sigma _{N}^{n} \nu _{{TH}}^{n} \left[ {\left( {n_{0} + \Delta n} \right)\left( {V_{D} + \Delta V_{D} } \right) - n_{{ND}} (V_{N} + \Delta V_{N} )} \right], \hfill \\ R_{{NA}} = \sigma _{A}^{n} \nu _{{TH}}^{n} \left[ {\left( {n_{0} + \Delta n} \right)\left( {V_{N} + \Delta V_{N} } \right) - n_{{AN}} (V_{A} + \Delta V_{A} )} \right], \hfill \\ R_{{ND}} = \sigma _{D}^{p} \nu _{{TH}}^{p} \left[ {\left( {p_{0} + \Delta p} \right)\left( {V_{N} + \Delta V_{N} } \right) - p_{{DN}} (V_{D} + \Delta V_{D} )} \right], \hfill \\ R_{{AN}} = \sigma _{N}^{p} \nu _{{TH}}^{p} \left[ {\left( {p_{0} + \Delta p} \right)\left( {V_{A} + \Delta V_{A} } \right) - p_{{NA}} (V_{N} + \Delta V_{N} )} \right], \hfill \\ \end{gathered}$$

5 $$\begin{gathered} n_{ND} = N_{C} \exp \left( { - \frac{{E_{N} }}{{k_{B} T}}} \right), \hfill \\ n_{AN} = N_{C} \exp \left( { - \frac{{E_{A} }}{{k_{B} T}}} \right), \hfill \\ p_{DN} = N_{V} \exp \left( { - \frac{{E_{G} - E_{N} }}{{k_{B} T}}} \right), \hfill \\ p_{NA} = N_{V} \exp \left( { - \frac{{E_{G} - E_{N} }}{{k_{B} T}}} \right), \hfill \\ \end{gathered}$$

where *G* is the generation term, *R*_*jj*_ represents recombination terms corresponding to different transition paths (e.g. *jj* is the *Donor Neutral* index that refers to transformation from V_D_ to V_N_ state), $\sigma_{j}^{i}$ is the capture cross-section of the *i*-type carrier by the V_j_ state, and $\nu_{TH}^{i}$ is the thermal velocity of different type carriers.

This phenomenology results from the V dopant substitution in the SiC. V contributes four outer shell electrons for bonding with nearest C atoms and has one free electron remaining in the *d* shell: V_Si_^4+^(3d^1^). This state is commonly referred as the neutral V state and will be labeled as V_N_(0) where 0 indicates impurity charge with respect to the SiC lattice. In 4H polytype of SiC, the V_N_ state has energy level located 1.6-eV below the conduction band, i.e. right at the mid-gap of the 3.2-eV band-gap. Addition of an extra electron to the outer shell of the neutral V produces the V_Si_^3+^(3d^2^) state which is referred as the acceptor state and will be labeled as V_A_. Increase of the total impurity charge increases Coulomb screening and reduces electron binding energy placing the V_A_ level a ~ 1-eV below the conduction band in 4H-SiC. Removal of an extra electron from the neutral V produces the V_Si_^5+^(3d^2^) state which is referred as the donor state and will be labeled as V_D_. This time the increase of electron binding energy pushes the V_D_ level into the valence band. Acceptor and donor levels, therefore, have opposite meaning as compared to shallow impurities where acceptor and donor states are associated with the valence (VB) and the conduction (CB) bands, respectively.

Representation (Fig. 2d) can be used to explain carrier recombination through amphoteric V centers. The main difference there is that blue arrows, free electrons and holes involved in the transitions are indicated by the blue and red circles, respectively. In contrast to conventional energy diagrams, the horizontal access now has meaning of the charge of the V state which has to be conserved in addition to carrier energy. Discrete transitions between different V charge states are indicated by +/0 and 0/− at the bottom of the diagram. Electron transitions from the V states into the valence band are also accompanied by the red arrows indicating simultaneous annihilation of holes. The diagram explains, for example, why it is not possible to have such transitions as the one involving electron and the V_A_ state (shown by the dashed gray arrows). The transition into the V_N_ state would violate charge conservation [V_A_(−) + e(−) = V_N_(0)], while transition into double charged acceptor [V_A_(−) − e(−) = V_A_(−−)] would violate energy conservation by pushing V into the conduction band where the V_A_(−−) state is predicted to exist.

High voltage electrical data was taken with the components immersed in Envirotemp FR3 oil. Main diagnostics consisted of a separately calibrated Magcap Engineering Type CT-103 current transformer and a properly compensated Tektronix P-6015 high voltage probe. Both were cross correlated to ensure accuracy of the measurement. Switching frequency data was taken by varying the repetition rate and noting the output level. This data was normalized to the lower frequency for comparison.

### Disclaimer

Reference herein to any specific commercial products, process, or service by trade name, trademark, manufacturer, or otherwise, does not necessarily constitute or imply its endorsement, recommendation, or favoring by the U.S. government or the Lawrence Livermore National Security, LLC. The views and opinions of authors expressed herein do not necessarily state or reflect those of the U.S. government or the Lawrence Livermore National Security, LLC, and shall not be used for advertising or product endorsement purposes.
